# Comparison of hidden blood loss and clinical efficacy of percutaneous endoscopic transforaminal lumbar interbody fusion and minimally invasive transforaminal lumbar interbody fusion

**DOI:** 10.1007/s00264-022-05485-z

**Published:** 2022-06-20

**Authors:** Meng Ge, Yuan Zhang, Hang Ying, Chenchen Feng, Yanlei Li, Jinlong Tian, Tingxiao Zhao, Haiyu Shao, Yazeng Huang

**Affiliations:** 1 Center for Plastic & Reconstructive Surgery, Department of Orthopedics, Zhejiang Provincial People’s Hospital (Affiliated People’s Hospital, Hangzhou Medical College), Shangtang Road 158#, Hangzhou, 310014 Zhejiang China; 2 Bengbu Medical College, Bengbu, China; 3 Center for General Practice Medicine, Department of Rheumatology and Immunology, Zhejiang Provincial People’s Hospital (Affiliated People’s Hospital, Hangzhou Medical College), Hangzhou, 310014 Zhejiang China; 4 Department of Orthopedics, Zhejiang Medical & Health Group Hangzhou Hospital, Hangzhou, China

**Keywords:** Hidden blood loss, Visual blood loss, Total blood loss, Percutaneous endoscopic transforaminal lumbar interbody fusion, Minimally invasive transforaminal lumbar interbody fusion

## Abstract

**Purpose:**

Hidden blood loss (HBL) is a growing area of interest for spinal surgeons. Simultaneously, spine surgeons’ pursuit of minimally invasive spine surgery has never ceased, as evidenced by the increasing number of articles comparing percutaneous endoscopic transforaminal lumbar interbody fusion (Endo-TLIF) and minimally invasive transforaminal lumbar interbody fusion (Mis-TLIF). However, there has been no comparison of HBL between Endo-TLIF and Mis-TLIF. This study aimed to compare HBL, visible blood loss (VBL), and total blood loss (TBL) following Endo-TLIF and Mis-TLIF and evaluate the clinical significance of these procedures.

**Methods:**

Between October 2017 and October 2019, 370 patients underwent lumbar interbody fusion at our institution and were followed up for at least 24 months. Our study included 41 Endo-TLIF and 43 Mis-TLIF cases. We recorded each patient’s age, height, weight, and haematocrit and calculated the TBL, which was used to indirectly obtain the HBL. Additionally, we compared the clinical outcomes of these two groups, including visual analogue scores for the lumbar spine and leg (VAS-Back; VAS-Leg), Oswestry Disability Index (ODI), Japanese Orthopaedic Association (JOA) scores, disease type, operative segment, and intervertebral fusion and complication rates.

**Results:**

Endo-TLIF had significantly lower HBL, VBL, and TBL values than Mis-TLIF (*P* < 0.05 for all). Although Endo-TLIF contained significantly less HBL than Mis-TLIF, the HBL to TBL ratio was statistically greater in Endo-TLIF (91%) than in Mis-TLIF (87%). Concerning clinical outcomes, VAS-Back, VAS-Leg, ODI, JOA, and Endo-TLIF demonstrated greater improvement rates than Mis-TLIF one week post-operatively. However, at the final follow-up, VAS-Back, VAS-Leg, ODI, and JOA scores all demonstrated a trend toward sustained improvement, with no statistically significant between-procedure difference. There were no statistically significant between-procedure differences in disease type, surgical segment, and complication or fusion rates.

**Conclusion:**

Endo-TLIF significantly reduced HBL, VBL, and TBL compared to Mis-TLIF and improved short-term clinical outcomes; however, long-term clinical outcomes and fusion rates remained comparable between the two groups, as did the incidence of peri-operative complications.

## Introduction

Lumbar spine fusion is an effective surgical technique for treating various lumbar spine disorders such as spinal stenosis and spondylolisthesis [1]. While open procedures such as posterior lumbar interbody fusion (PLIF) and transforaminal lumbar interbody fusion (TLIF) remain effective, many patients, particularly older adults, cannot tolerate them because of their numerous associated complications [2]. Therefore, a minimally invasive procedure was required in the form of advancements in minimally invasive spine surgery (MISS) [3, 4]. Minimally invasive lumbar interbody fusion (Mis-TLIF) is a viable treatment for degenerative spinal conditions, as it is less invasive than open surgery, results in significantly less intra-operative bleeding, and requires less post-operative recovery time [4]. As optical techniques and specialised instruments advance, minimally invasive endoscopic surgery is gaining popularity. Percutaneous endoscopic transforaminal lumbar interbody fusion (Endo-TLIF) has recently grown in popularity amongst spine surgeons because of its small incision, safety and effectiveness, favourable post-operative outcomes, low complication rate, and ability to relieve pain in older adults who cannot tolerate open surgery [5–8]. Numerous reports have compared Endo-TLIF and Mis-TLIF, examining the two procedures from various angles to arrive at a less invasive procedure [3, 4, 9, 10].

Sehat et al. introduced the concept of hidden blood loss (HBL), demonstrating that it accounted for 26 and 49% of the total blood loss (TBL) following total knee and hip arthroplasty, respectively, but was frequently overlooked by surgeons because of its invisibility [11]. HBL has gradually gained the attention of spine surgeons as a result of this literature [12, 13]. A growing body of literature indicates that HBL increases TBL and post-operative complications, such as prolonged recovery and hospitalisation, and the likelihood of other cardiovascular and cerebrovascular accidents increases with increased bleeding volume [14–16]. Therefore, HBL must be strictly managed to minimise post-operative complications.

While there is a substantial body of literature comparing Endo-TLIF and Mis-TLIF procedures, to our knowledge, no comparison has been made on the critical factors of HBL, rendering the comparison of these two procedures incomplete and resulting in a significant shortcoming in the management of patients following MISS. Therefore, we included the critical factor of HBL in this retrospective study comparing the two procedures, Endo-TLIF and Mis-TLIF, to provide a more comprehensive comparison of the two procedures and a theoretical reference for the MISS procedure’s post-operative management.

## Materials and methods

### Clinical information

**Fig. 1:**
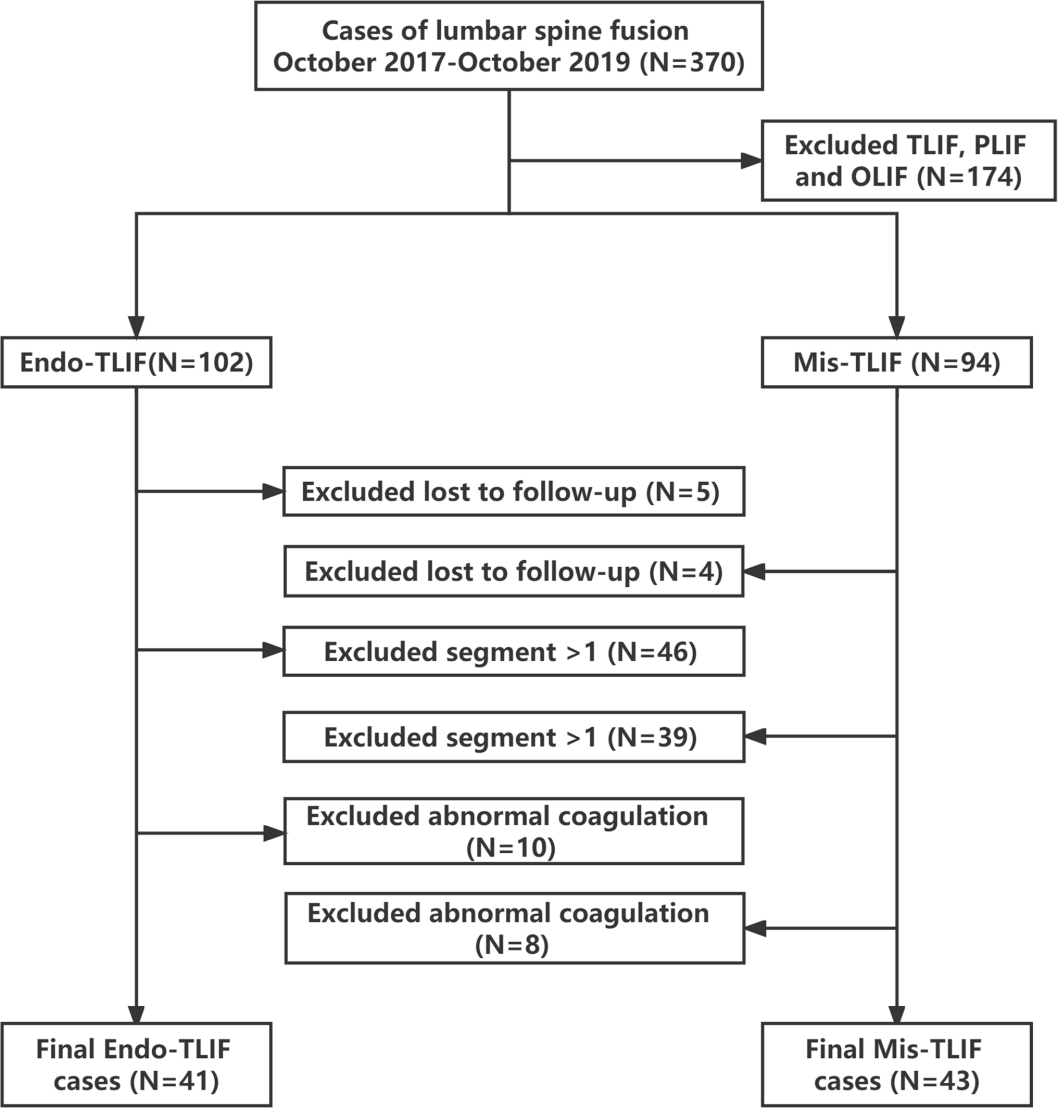
Case selection process

Between October 2017 and October 2019, 370 patients underwent lumbar interbody fusion at our institution. A total of 174 patients who underwent TLIF, PLIF, or oblique lateral interbody fusion were excluded. Five patients were lost to follow-up in the Endo-TLIF group, 46 cases of segment > 1, and 10 cases of abnormal coagulation, all of which were excluded. Four patients were lost to follow-up in the Mis-TLIF group, 39 in the segment > 1, and eight cases of abnormal coagulation, all of which were excluded. Finally, we included 84 cases, 41 and 43 Endo- and Mis-TLIF, respectively (Fig. 1).

The following inclusion criteria were used: (1) patients aged 18 years or older; (2) lumbar spinal stenosis; (3) mild lumbar spondylolisthesis (Meyerding grades I and II); (4) disc herniation with single-segment lumbar instability; (5) recurrent lumbar disc herniation; (6) patients whose symptoms did not resolve or worsened after conservative treatment for ≥ six months; and (7) patients who were followed up for ≥ 24 months.

The exclusion criteria were as follows: (1) patients who could not tolerate surgery because of severe systemic diseases; (2) segment > 1; (3) severe lumbar spondylolisthesis (Meyerding grade III); (4) haematologic-related diseases; (5) severe heart, brain, kidney, or other diseases; and (6) history of lumbar spine surgery, severe osteoporosis, lumbar spine tumour, tuberculosis, or infection. Additionally, all patients in this study underwent surgery performed by the same senior surgeon and were at the plateau of the surgeon’s learning curve. The hospital’s institutional ethics committee approved this study and all participants provided written informed consent.

### Endo-TLIF

After general anaesthesia, the patients were placed in the prone position, with the sides of the abdomen elevated to reduce lumbar lordosis. C-arm fluoroscopy was used to locate the space of lesion segment, and then, the position of the surgical channel of intervertebral foramen and the position of the skin incision of pedicle screw were marked. After routine disinfection of the skin, a sterile sheet was laid and a protective film was affixed to the incision. And the needle entry point was determined (a longitudinal incision of 10 mm was made 5 cm from the posterior midline, centred on the space of lesion segment, which is a combination of decompression, fusion, and percutaneous pedicle screw placement; the length of this incision can be fine-tuned in order to facilitate decompression parallel to the intervertebral space and the placement of the interbody Fusion Cage). The guide needle was then placed, and after confirming that the fluoroscopy position of the C-arm is satisfactory, the gradually expanding catheter was used to separate the soft tissue, and then, the working channel of the spinal endoscopy was placed. When the C-arm fluoroscopy results showed that the position of the working channel was satisfactory, the light source and monitor were connected. The joint capsule was treated with endoscopic electrocoagulation. Progressively, the superior and inferior synovial joints and part of the lamina of the vertebra of the lesion space were excised using a circular saw between the superior border of the inferior vertebral arch and the inferior border of the superior vertebral arch. The nerve roots and dural cyst of lesion segment were exposed after excision of the ligamentum flavum, the nerve root was decompressed after being protected by the working cannula, the annulus fibrosus was cut, and the intervertebral space was cleared. After removing the residual annulus fibrosus and nucleus pulposus, scrape the upper and lower endplate of the lesion intervertebral space with a reamer. An appropriately sized test mould was selected when the end plate was properly processed. The bone was removed from the contralateral posterior superior iliac spine using an incision in the contralateral pedicle screw channel and prepared into a bone pellet then, which was filled into the interbody Fusion Cage and placed into the lesion segment intervertebral space. The nerve root on the affected side of the lesion segment was re-examined, the working channel was withdrawn when adequate decompression was demonstrated, and C-arm fluoroscopy indicated that the interbody Fusion Cage was in a suitable position. The skin was then incised approximately 10 mm at the bilateral pedicle screw projection in the upper and lower cones of the lesion segment, and pedicle screws and bilateral connecting rods were placed percutaneously. After C-arm fluoroscopy indicated that the screw channel and rods were well positioned, the skin was sutured and covered with a sterile dressing.

### Mis-TLIF

The patients were placed in the prone position after general anaesthesia, the lesion segment intervertebral space was located using C-arm fluoroscopy, and the incision site was marked on the skin. After routine disinfection and sterile sheeting, a protective film strip was applied to the incision site. The incision was located in the paracentral part of the spinous process of the lesion segment and was approximately 3 cm long on both the right and left sides. The skin and subcutaneous tissues were incised, and the small joints and transverse process roots on both sides of the lesion segment were exposed through the muscle space approach. Pins were placed on both sides of the arch using the conventional screw placement method.

On anteroposterior and lateral fluoroscopy, the segments were correctly positioned, the guide needles were correctly positioned and oriented, and the four pedicle screw paths were prepared with tap. The screw path on the lesion side was closed with bone wax, the pedicle screws were screwed into the contralateral screw paths, and a shaped fixation bar was mounted and two nuts were fitted (no tightening). The sacrospinous muscle on the affected side was stripped to expose the lamina of the vertebra and the small joint of the lesion segment; then, the expansion channel was placed at the small joint and fixed with a free arm.

The ligamentum flavum was removed after excision of the inferior articular process of the superior vertebral body and the superior articular process of the inferior vertebral body using a bone knife. With the nerve roots and dural cyst protected, the annulus fibrosus was circumferentially dissected from the affected side of the intervertebral space. The disc nucleus pulposus was removed, and the intervertebral space was cleared to remove the residual annulus fibrosus and nucleus pulposus. The adjacent vertebral endplate cartilage was then scraped, the intervertebral space was gradually opened by using the intervertebral disc opener, and the intervertebral space was loosened. The interbody Fusion Cage (with autologous decompression bone) was placed anteriorly in the intervertebral space after selecting a suitably sized cage using a test mould. Subsequently, the pedicle screws and shaped titanium rods were fitted on the affected side, the fixation nuts were tightened, and then, all the fixing nuts were tightened on the opposite side. After C-arm fluoroscopy showed that the internal fixation screws and interbody Fusion Cage were well positioned, the incision was repeatedly irrigated, and the lumbodorsal fascia, subcutaneous tissue, and skin were sutured in sequence. The incision was dressed with a sterile dressing.

### Post-operative management

Both patient groups were routinely bedridden post-operatively and received the same rehydration regimen, including low doses of hormones and pain medications. Anticoagulants were not administered to any patients because they were bedridden for a short time. All patients were routinely treated with pneumatic compression of the extremities and were instructed to turn and lift their legs in bed to avoid thrombosis. These exercises are also beneficial for assisting patients in getting out of bed post-operatively. We routinely reviewed all patients using radiography, computed tomography, and magnetic resonance imaging on the first post-operative day. When all examination findings were normal and wound conditions were met, the patients were instructed to walk under the protection of the brace.

### Calculation of HBL

HBL is equal to TBL minus visual blood loss (VBL) plus transfusion. Therefore, to calculate HBL, we needed to calculate the TBL and VBL. For calculating TBL, the formula of Gross et al. states that TBL (mL) = patient’s blood volume (PBV; L) × (Hct-pre − Hct-post) / (Hct-pre + Hct-post) / 2 × 1000 (Hct-pre means pre-operative Hct, and Hct-post means post-operative Hct). According to the formula guiding the patient’s haematocrit (Hct), we also chose the pre-operative and Hct values after blood volume stabilisation for two to three days post-operatively [17]. For PBV, Nadler et al. suggested that it can be calculated based on the patient’s sex, height, and weight, that is, PBV (L) = *k*1 × height (m)^3^ + *k*2 × weight (kg) + *k*3, where *k*1 = 0.3669, *k*2 = 0.03219, and *k*3 = 0.6041 for men, and *k*1 = 0.3561, *k*2 = 0.03308, and *k*3 = 0.1833 for women [18].

Because no drains were placed in any patients post-operatively, VBL ≈ intra-operative blood loss. Intra-operative blood loss included the weight of the blood in the suction bottle (subtracting the irrigation fluid used during surgery) and in the gauze and gauze strips (subtracting the dry gauze and dry gauze strips weight used during surgery), and no patients were transfused intra-operatively or post-operatively, so HBL = TBL − intra-operative blood loss. From the above statement, to obtain the HBL value, we only needed to calculate the TBL based on the change in Hct and calculate the VBL based on the intraoperative blood loss. The difference between TBL and intraoperative blood loss was due to HBL.

### Statistical analysis

The data were analysed using SPSS v26.0 for Windows (IBM Corp., Armonk, NY, USA). All values are expressed as mean ± standard deviation. Continuous variables including age, body mass index, visual analogue scale (VAS) for the back (VAS-Back) and legs (VAS-Leg), the Oswestry Disability Index (ODI) and Japanese Orthopaedic Association (JOA) scores, preoperative Hct, PBV, surgical duration, TBL, VBL, HBL, and the ratio of HBL were screened using independent sample *t*-tests. Categorical data, including sex, disease aetiology, surgical segments, interbody fusion, and complications, were screened using the chi-squared test. Statistical significance was defined as *P* < 0.05.

## Results

For pre-operative baseline data, there was no statistically significant difference between the two procedures (*P* > 0.05; Table 1). The HBL in the Endo-TLIF group was significantly smaller than that in the Mis-TLIF group (Endo-TLIF: 717.9 ± 220.1; Mis-TLIF: 942.3 ± 219.1; *P* < 0.001). TBL and VBL were significantly less in the Endo-TLIF group compared with those in the Mis-TLIF group (Endo-TLIF: 785.8 ± 243.1 and 69.5 ± 30.3 mL, Mis-TLIF: 1087.1 ± 250.9 mL and 144.8 ± 37.2 mL, both *P* < 0.001). There was also a significant difference between the two groups regarding HBL as a percentage of TBL (Endo-PLIF, 91%; Mis-TLIF, 87%; *P* < 0.001; Table 2).

**Table 1:** Comparison of pre-operative baseline data between Endo-TLIF and Mis-TLIF

	Endo-TLIF	Mis-TLIF	*P* value
Age	59.6 ± 7.6	62.7 ± 10.4	0.184
Gender (male/female)	21/20	19/24	0.519
BMI (kg/m^2^)	24.0 ± 2.8	25.2 ± 2.6	0.057
Disease aetiology			0.895
Spinal stenosis	19	20	
Spondylolisthesis	5	7	
Disc herniation with segmental instability	14	12	
Recurrent lumbar disc herniation	3	4	
Surgical segments			0.810
L3/4	1	2	
L4/5	33	35	
L5/S1	7	6	
VAS (Back)	6.5 ± 0.6	6.4 ± 0.7	0.633
VAS (Leg)	7.1 ± 0.6	3.1 ± 0.4	0.988
ODI (%)	44.4 ± 3.3	44.5 ± 2.3	0.875
JOA	10.0 ± 1.8	10.4 ± 1.7	0.215
Preoperative Hct	0.407 ± 0.4	0.412 ± 0.0	0.539
PBV(L)	4.15 ± 0.6	4.210 ± 0.6	0.659

**Table 2:** Comparison of peri-operative volume loss of blood between Endo-TLIF and Mis-TLIF

	ENDO-TLIF	MIS-TLIF	*P* value
Total blood loss (mL)	785.8 ± 243.1	1087.1 ± 250.9	< 0.001
Visible blood loss (mL)	69.5 ± 30.3	144.8 ± 37.2	< 0.001
Hidden blood loss (mL)	717.9 ± 220.1	942.3 ± 219.1	< 0.001
Ratio of hidden blood loss (%)	91%	87%	< 0.001

The surgical duration was longer in the Endo-TLIF than in the Mis-TLIF group (206.5 ± 12.4 vs 130.5 ± 14.6 minutes, *P* < 0.001). One week post-operatively, the Endo-TLIF group had lower VAS-Back, VAS-Leg, and ODI scores than the Mis-TLIF group (VAS-Back: 1.5 ± 0.6 vs 2.5 ± 0.6, *P* < 0.001; VAS-Leg: 1.9 ± 0.6 vs 3.1 ± 0.4; ODI: 18.1 ± 4.1 vs 22.0 ± 2.8, *P* < 0.001), and the JOA scores one week post-operatively were significantly higher in the Endo-TLIF group than in the Mis-TLIF group (21.0 ± 1.6 vs 18.2 ± 1.4, *P* < 0.001). All patients were followed up regularly for at least two years, and the VAS-Back, VAS-Leg, and ODI and JOA scores were statistically insignificant in both groups at the final follow-up (VAS-Back: 0.15 ± 0.358 vs 0.28 ± 0.5, *P* = 0.201; VAS-Leg: 0.29 ± 0.5 vs 0.37 ± 0.7, *P* = 0.814; ODI: 10.9 ± 2.9 vs 10.67 ± 1.7, *P* = 0.344; JOA: 24.8 ± 2.3 vs 24.5 ± 1.8, *P* = 0.336). There was no significant between-group difference in the type of disease or surgical segments. According to the Bridwell criteria [7], the interbody fusion rate was essentially the same between the two groups (97.6% in the Endo-TLIF group and 95.3% in the Mis-TLIF group, *P* = 0.585). One patient in the Endo-TLIF group developed symptoms of lower extremity radiation pain due to improper pedicle screw placement. Two patients in the Mis-TLIF group experienced post-operative complications, including haematoma formation in one patient and cerebrospinal fluid dew in the other, with no significant between-group differences in complication rates (Endo-TLIF, 2.4%; Mis-TLIF, 4.7%; *P* = 0.585). All three patients completely recovered after conservative treatment or haematoma removal by percutaneous endoscopic lumbar discectomy and screw tract adjustment. No organic-related complications such as nerve injury or implant loosening were detected during the follow-up period (Table 3).

**Table 3:** Comparison of intra-operative and post-operative data between Endo-TLIF and Mis-TLIF

	Endo-TLIF	Mis-TLIF	*P* value
Operative time (min)	206.5 ± 12.4	130.5 ± 14.6	< 0.001
Interbody fusion (grade I/II)	1/40	2/41	0.585
Complication (yes/no)	1/40	2/41	0.585
VAS (back)			
One week post-operation	1.5 ± 0.6	2.5 ± 0.6	< 0.001
Final follow-up	0.15 ± 0.358	0.28 ± 0.5	0.201
VAS (leg)			
One week post-operation	1.9 ± 0.6	3.1 ± 0.4	< 0.001
Final follow-up	0.29 ± 0.5	0.37 ± 0.7	0.814
ODI (%)			
One week post-operation	18.1 ± 4.1	22.0 ± 2.8	< 0.001
Final follow-up	10.9 ± 2.9	10.67 ± 1.7	0.344
JOA			
One week post-operation	21.0 ± 1.6	18.2 ± 1.4	< 0.001
Final follow-up	24.8 ± 2.3	24.5 ± 1.8	0.336

## Discussion

HBL has been shown to increase TBL, exacerbate post-operative haemoglobin decline, increase transfusion requirements, and, if not managed appropriately, may result in peri-operative complications such as delayed wound healing, increased infection risk, and prolonged post-operative recovery time [19]. HBL has become a growing concern amongst spine surgeons since the year 2000. Lei et al. reported that the mean HBL during posterior lumbar fusion surgery was 449 ± 191 mL, with 44.2 ± 16.6% in HBL and TBL, respectively [16]. According to Ju et al., HBL accounted for an average of 39.2% of the actual TBL during anterior lumbar interbody fusion [13]. As MISS becomes more prevalent, researchers are becoming aware of the role of HBL; Wu et al. reported that the mean HBL during percutaneous kyphoplasty was 282 ± 163 mL [20], while Zhou et al. reported that the mean HBL during Mis-TLIF was 488.4 ± 294.0 mL or 52.5% of the TBL [21].

While numerous articles have been published on HBL, no study has examined this issue in Endo- and Mis-TLIF. This article introduces this issue for the first time and compares Endo- and Mis-TLIF in terms of critical factors and clinical outcomes. This article aimed to recommend a less invasive procedure for spine surgeons. According to the results, Endo-TLIF had a significantly lower HBL than Mis-TLIF and lower VBL and TBL values. This may be attributed to the improved soft tissue protection provided by Endo-TLIF. The Endo-TLIF field is accessed via a small 1.5-cm incision, and all pedicle screws are placed percutaneously, whereas the Mis-TLIF field is accessed via bilateral skin incisions, and all pedicle screws are placed via the intramuscular approach.

However, the percentage of HBL in this study was higher than that reported by other researchers [14, 22], most likely because no drainage tube was placed following either procedure, resulting in a higher percentage of HBL as a proportion of TBL. According to our results, Endo-TLIF resulted in significantly less blood loss than Mis-TLIF, but the operative time was longer, suggesting that operative time may not be a risk factor for HBL; the details will need to be clarified in subsequent studies, but Shen does not believe that the time of surgery is a risk factor for HBL [23]. At one week post-operatively, Endo-TLIF was more effective than Mis-TLIF in pain relief and patient function improvement, most likely because Mis-TLIF causes short-term pain and limited mobility, and it can also be inferred that Endo-TLIF provides better soft tissue protection than Mis-TLIF.

Endo-TLIF is a less invasive procedure than Mis-TLIF. Due to an increasingly ageing society, the number of older adults with compromised basic vital signs is gradually increasing [24], and peri-operative bleeding must be strictly controlled to avoid adverse events. Therefore, Endo-TLIF should be recommended for these patients to further reduce peri-operative bleeding and the incidence of adverse events.

Although we obtained relatively satisfactory results, this study has some limitations. First, this was a single-centre retrospective study with a small sample size and a single surgical segment. Second, the calculation of intra-operative blood loss was not very rigorous, as some irrigation fluid did not reach the drainage bottle, resulting in underestimating intra-operative blood loss. Third, we used the Hct at two to three days post-operatively as a reference [25], but haemodynamic stability at two to three days post-operatively is still unknown, and thus, the amount of HBL may be under- or overestimated. Therefore, future studies should use larger multicentre samples and include patients with multiple surgical segments. In addition, accurate methods for assessing post-operative fluid balance and haemodynamics are required.

The HBL, VBL, and TBL levels in the Endo-TLIF group were significantly lower than those in the Mis-TLIF group, and the Endo-TLIF group had significantly better clinical outcomes during short-term follow-up. There was no significant between-group difference in clinical outcomes at the final follow-up.

## Data Availability

The datasets used or analysed during the current study are available from the corresponding author on reasonable request.
